# Comprehensive proteomics of CSF, plasma, and urine identify DDC and other biomarkers of early Parkinson’s disease

**DOI:** 10.1007/s00401-024-02706-0

**Published:** 2024-03-11

**Authors:** Jarod Rutledge, Benoit Lehallier, Pardis Zarifkar, Patricia Moran Losada, Marian Shahid-Besanti, Dan Western, Priyanka Gorijala, Sephira Ryman, Maya Yutsis, Gayle K. Deutsch, Elizabeth Mormino, Alexandra Trelle, Anthony D. Wagner, Geoffrey A. Kerchner, Lu Tian, Carlos Cruchaga, Victor W. Henderson, Thomas J. Montine, Per Borghammer, Tony Wyss-Coray, Kathleen L. Poston

**Affiliations:** 1 Department of Genetics, Stanford University School of Medicine, Stanford University, Stanford, CA USA; 2 Department of Neurology and Neurological Sciences, Stanford University School of Medicine, Stanford University, Stanford, CA USA; 3 Department of Psychiatry, Washington University in St Louis, St Louis, MO USA; 4 NeuroGenomics and Informatics Center, Washington University School of Medicine, St. Louis, MO USA; 5 Department of Clinical Epidemiology, Aarhus University, Aarhus, Denmark; 6 Wu Tsai Neurosciences Institute, Stanford University School of Medicine, Stanford, CA USA; 7 Translational Neuroscience, Mind Research Network, Albuquerque, NM USA; 8 Department of Psychology, Stanford University School of Medicine, Stanford University, Stanford, CA USA; 9 Roche Medical, Basel, Switzerland; 10 Department of Biomedical Data Science, Stanford University School of Humanities and Sciences, Stanford University, Stanford, CA USA; 11 Department of Epidemiology and Population Health, Stanford University, Stanford, CA USA; 12 Department of Pathology, Stanford University School of Medicine, Stanford University, Stanford, CA USA; 13 Department of Nuclear Medicine and PET, Aarhus University Hospital, Aarhus, Denmark; 14 The Knight Initiative for Brain Resilience, Stanford University, Stanford, CA USA; 15 Department of Neurosurgery, Stanford University School of Medicine, Stanford University, Stanford, CA USA

## Abstract

Parkinson’s disease (PD) starts at the molecular and cellular level long before motor symptoms appear, yet there are no early-stage molecular biomarkers for diagnosis, prognosis prediction, or monitoring therapeutic response. This lack of biomarkers greatly impedes patient care and translational research—l-DOPA remains the standard of care more than 50 years after its introduction. Here, we performed a large-scale, multi-tissue, and multi-platform proteomics study to identify new biomarkers for early diagnosis and disease monitoring in PD. We analyzed 4877 cerebrospinal fluid, blood plasma, and urine samples from participants across seven cohorts using three orthogonal proteomics methods: Olink proximity extension assay, SomaScan aptamer precipitation assay, and liquid chromatography–mass spectrometry proteomics. We discovered that hundreds of proteins were upregulated in the CSF, blood, or urine of PD patients, prodromal PD patients with DAT deficit and REM sleep behavior disorder or anosmia, and non-manifesting genetic carriers of LRRK2 and GBA mutations. We nominate multiple novel hits across our analyses as promising markers of early PD, including DOPA decarboxylase (DDC), also known as l-aromatic acid decarboxylase (AADC), sulfatase-modifying factor 1 (SUMF1), dipeptidyl peptidase 2/7 (DPP7), glutamyl aminopeptidase (ENPEP), WAP four-disulfide core domain 2 (WFDC2), and others. DDC, which catalyzes the final step in dopamine synthesis, particularly stands out as a novel hit with a compelling mechanistic link to PD pathogenesis. DDC is consistently upregulated in the CSF and urine of treatment-naïve PD, prodromal PD, and GBA or LRRK2 carrier participants by all three proteomics methods. We show that CSF DDC levels correlate with clinical symptom severity in treatment-naïve PD patients and can be used to accurately diagnose PD and prodromal PD. This suggests that urine and CSF DDC could be a promising diagnostic and prognostic marker with utility in both clinical care and translational research.

**Supplementary Information:**

The online version contains supplementary material available at 10.1007/s00401-024-02706-0.

## Introduction

Parkinson’s disease (PD) is characterized by selective neuronal degeneration of the substantia nigra and the accumulation of predominantly neuronal alpha-synuclein protein aggregates termed Lewy bodies [2]. The defining histopathologic feature of the disease is the loss of dopaminergic neurons in the substantia nigra but can also include noradrenergic neurons in the locus coeruleus [11] and serotonergic neurons in the dorsal raphe nuclei [16]*.* PD and other neurodegenerative diseases which present with parkinsonian-like symptoms have a high rate of misdiagnosis, particularly at initial symptom presentation and there are few molecular biomarkers to aid diagnosis and monitor patient care [65]. Newly developed alpha-synuclein seeding assays [36, 43, 47, 48, 55] (αSyn-SAA) can identify misfolded alpha-synuclein in cerebrospinal fluid (CSF) and can be used as a proxy for the presence of Lewy body pathology in the brain to assist diagnosis of PD and other synucleopathies. While a major advance, αSyn-SAAs has multiple limitations. The αSyn-SAA has limited utility for some patients with LRRK2 mutations and others who have nigrostriatal neurodegeneration without Lewy bodies at autopsy [52, 55]. Further, αSyn-SAA does not provide information about disease severity or progression which remains an important unmet need in PD research and care. It also remains unclear how sensitive and generalizable these tests will be for early detection of PD before emergence of motor symptoms [55]. Lastly, αSyn-SAA requires a lumbar puncture and a complex custom incubation assay which takes many days, posing challenges for widespread use in screening, clinical trials, and routine care.

Thus, there remains an urgent need for additional minimally invasive biomarkers which reflect the early underlying neurodegenerative processes in PD to augment diagnosis and to assess disease severity, forecast disease progression, and monitor response to therapeutics [37, 44, 56]. In particular, damage to dopaminergic neurons begins many years before the development of clinical motor symptoms, and biomarkers which can detect this damage in patients in the asymptomatic phase are urgently needed [58]. In Alzheimer’s disease (AD) research, the development of such quantitative CSF and blood plasma biomarkers which reflect early pathologic events in the brain and which correlate with symptoms and predict future decline enabled the development of new disease-modifying therapies [3, 9, 57]. Similarly, recent drug approvals for amyotrophic lateral sclerosis were based on a reduction in plasma neurofilament light [31], a biomarker of axonal injury and neurodegeneration.

**Fig. 1:**
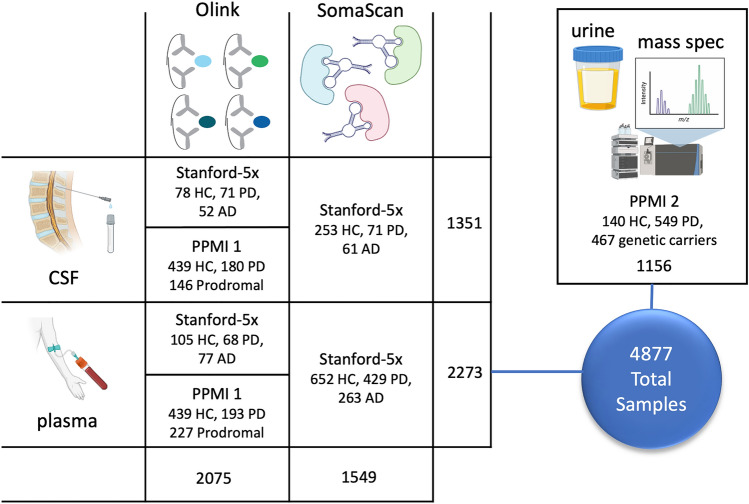
Illustration of the study design. Three proteomics platforms (Olink, SomaScan, LC–MS/MS) were used to analyze proteomics from three different biofluids (CSF, plasma, urine) in multiple cohorts (Stanford-5x, PPMI 1, and PPMI 2). CSF and plasma were collected from Stanford-5x and PPMI 1, while urine was collected from PPMI 2. Inset within each square are the number of samples from healthy controls (HC), Parkinson’s disease (PD), and AD-spectrum (AD) participants. The total sample numbers are summed across the rows and columns, providing information on the total number of samples run with each assay and biofluid

Quantitative proteomics has recently been used to develop disease-specific protein signatures as diagnostic biomarkers and holds great promise to enhance our current understanding of the molecular mechanisms underlying neurological diseases. In this study, we integrated CSF, plasma, and urine proteomics from three orthogonal proteomics assays in multiple independent human cohorts (Fig. 1) to identify disease-specific protein signatures of early PD that correlated with observed clinical severity and could distinguish PD participants from cognitively normal individuals and Alzheimer’s disease participants.

## Methods

### Stanford research cohorts

We included participants from five different studies of aging and neurodegeneration at Stanford University: (1) Biomarkers in PD Study (BPD), (2) the Pacific Udall Center (PUC), (3) Stanford Alzheimer’s Disease Research Center (ADRC), (4) Stanford Center for Memory Disorders Cohort Study (SCMD), and (5) Stanford Aging and Memory Study (SAMS). The combined cohort is, Hereafter, referred to as Stanford-5x. Data were collected between 2012 and 2018. Inclusion criteria for these analyses were (1) ages between 40 and 90 years (2) English or Spanish fluency for comprehensive neuropsychological testing, and (3) no contraindications to lumbar puncture. All participants provided written informed consent to participate in the parent studies following protocols approved by the Stanford Institutional Review Board.

A consensus panel consisting of one board-certified movement disorders neurologist or behavioral neurologist, one board-certified neuropsychologist, and other study personnel adjudicated the diagnosis for each participant. PD diagnosis was based on UK PD Society Brain Bank clinical diagnostic criteria [23]. We defined Early PD as participants with less than three years since diagnosis at the time of CSF collection. Participants on the AD spectrum (AD-s) included those with dementia or mild cognitive impairment likely due to AD based on the NIH Alzheimer’s Disease Diagnostic Guidelines [30, 40]. Participants with mild cognitive impairment who have decreased CSF Aβ-42 concentration, are more likely to have cognitive impairments due to AD [18]. To exclude participants without AD from the AD-s group, we excluded mild cognitive impairment participants who had CSF Aβ-42 concentration more than two standard deviations above the mean in AD [54, 62]. Participants with PD include those with no cognitive impairment, those with mild cognitive impairment [24] and those with dementia due to PD. Healthy controls (HC) were older individuals without a neurological diagnosis adjudicated as cognitively normal for age at the consensus meeting.

Some participants were excluded from the current study because of incomplete metadata on age, sex, or disease status. After filtering based on inclusion criteria, 201 CSF samples (71 PD, 78 HC, and 52 AD-s) and 250 blood plasma samples (68 PD, 105 HC, and 77 AD-s) were sent to Olink Proteomics AB (http://www.olink.com) for proteomics using a multiplex proximity extension assay [1], described in detail in a separate methods section 385 CSF samples (71 PD, 253 HC, 61 AD-s) and 1164 plasma samples (249 PD, 652 HC, 263 AD-s) were sent to SomaLogic Inc. (somalogic.com) for proteomics using a multiplex aptamer affinity assay, described in detail in a separate methods section.

#### Neurologic, motor and cognitive assessments

All participants completed a general neurological exam. PD participants completed the Movement Disorders Society-Unified Parkinson’s Disease Rating Scale (MDS-UPDRS III) [13] in the Off- and On-medication states, according to published criteria [34]. We calculated the Levodopa Equivalent Daily Dose (LEDD) using previously reported conversion factors [63, 66].

Global cognitive function was assessed using the Montreal Cognitive Assessment (MoCA) [33] in the ADRC, PUC and BPD, and the Mini-Mental State Exam [32] in the SAMS and SCMD studies. PD participants underwent neuropsychological testing in the on-medication state in order to assess cognitive function without interference by motor deficits.

#### CSF collection and assessment

A neurologist performed a lumbar puncture to collect CSF samples according to procedures standardized across all Stanford-5x cohorts [10]. Briefly, a 20–22 G spinal needle was inserted in the L4–L5 or L5–S1 interspace and CSF was collected in polypropylene tubes. The tubes were immediately frozen at − 80 °C in a centralized freezer in the Neuropathology Core of the Stanford ADRC.

### PPMI cohort

Data used in the preparation of this article were obtained on August 8, 2023, from the Parkinson’s Progression Markers Initiative (PPMI) database (http://www.ppmi-info.org/access-dataspecimens/download-data), RRID:SCR 006431. For up-to-date information on the study, visit http://www.ppmi-info.org.

The Parkinson's Progression Markers Initiative (PPMI) is an ongoing observational, international study conducted in the United States, Europe, Israel, and Australia. The study has enrolled approximately 4000 participants to date which includes healthy adults (HC), de novo PD, prodromal (age 60 or older with DAT deficit and REM sleep behavior disorder (RBD) or hyposmia), and non-manifesting LRRK2 and GBA carrier participants. Participants undergo extensive clinical assessment, imaging, and molecular phenotyping. Here, we have used data from two PPMI sub-studies: project 190 and project 196. Both studies were performed by industry research groups in collaboration with PPMI and shared in the online PPMI portal as part of the PPMI data use agreement. While each study uses samples from PPMI participants, they have few overlapping participants (42 PD, 92 HC, and 5 genetic carriers) and no overlapping samples since they focused on different tissues and proteomics methods. We have labeled them as PPMI 1 (project 196) and PPMI 2 (project 190) in the main text for clarity.

#### PPMI patient nomenclature

PPMI has recruited multiple participant groups and a detailed description of groups is available at https://www.ppmi-info.org/study-design/study-cohorts/.

PD cohort: all participants of the PD cohort have a clinical diagnosis of PD and a positive dopamine transporter (DAT) SPECT. The PD cohort is comprised of several subgroups, which include the following key inclusion criteria:

De novo PD: people with untreated PD and within 2 years of diagnosis at enrollment. The initial phase of PPMI enrolled 423 untreated PD participants.

Genetic PD: people with PD and pathogenic genetic variant(s) in LRRK2 or GBA, within 7 years of diagnosis. Treatment with medication was allowed at enrollment, therefore, some of the participants were medicated and some were still de novo at baseline visit. The initial phase of PPMI enrolled 294 genetic PD participants (across variants).

Prodromal cohort: participants who are at risk of Parkinson’s based on clinical features, genetic variants, or other biomarkers.

Prodromal (RBD or anosmia): the initial phase of PPMI enrolled 67 prodromal volunteers age 60 or older with DAT deficit and REM sleep behavior disorder (RBD) or hyposmia.

Prodromal (non-manifesting genetic carriers): the initial phase of PPMI enrolled 445 prodromal volunteers with a genetic risk variant (SNCA, LRRK2, GBA).

#### Project 196 (PPMI 1)

A study document can be found at https://ida.loni.usc.edu/download/files/study/4bb082de-fa77-40ce-9dc5-494ec7fc0a1f/file/ppmi/PPMI_Project_196_Methods_Explore_20221212.pdf.

Briefly, project 196 is a longitudinal Olink proteomics study of de-novo PD and non-genetic prodromal participants age 60 or older with RBD or hyposmia and DAT deficit, with repeat sampling over 4 years. Data from study 196 were downloaded, totaling 924 CSF samples and 1160 plasma samples. The data are provided in Olink NPX units, a normalized arbitrary unit derived from sequence counts. The Olink NPX calculation and QC process are described in a separate section for clarity. Project 196 documents describe the data generation occurred in two separate experimental batches more than one year apart, so a batch effect analysis using principal component analysis (PCA) was performed. A batch effect corresponding to the experimental batch was observed via PCA. The study design utilized bridging samples to allow for correction of batch effects. We noted 89 bridging samples in both CSF and plasma datasets. We performed a batch correction based on PLATEID, using the removeBatchEffect function in the limma package for R 4.0.3. We confirmed via PCA that this removed the experimental batch effect. After batch correction, NPX values for replicate bridging samples were averaged to avoid duplicates in downstream analysis. The dataset was then further filtered to include only samples in which DDC was detected, since a fraction of samples were missing data from the Olink Cardiometabolic panel which contains DDC. After merging the filtered and QC’d data with participant level metadata, there were 765 CSF samples from 257 participants, consisting of 180 PD, 439 HC, and 146 prodromal samples. There were 859 plasma samples from 274 participants, consisting of 193 PD, 439 HC, and 227 prodromal samples. These data were used for all downstream CSF and plasma analysis in PPMI. For analysis of baseline samples, there were 243 CSF samples (69 de novo PD, 130 HC, 44 prodromal) and 262 plasma samples (74 de novo PD, 130 HC, 58 prodromal).

#### Project 190 (PPMI 2)

A study document can be found at the PPMI website, https://ida.loni.usc.edu/download/files/study/0a00d6e1-cd85-4340-9913-b2436a94acc1/file/ppmi/PPMI_190_Methods_Targeted_Untargeted_MS-based_proteomics_of_urine.pdf.

Briefly, project 190 is an LC–MS/MS proteomics study of urine samples from PD and non-manifesting GBA and LRRK2 mutation carriers, with a small amount of longitudinal data in LRRK2 carriers. Data from study 190 was provided in a normalized and QC’d form. After merging with patient-level metadata there were 1156 samples from 983 participants, consisting of 549 PD, 140 HC, and 467 non-manifesting carrier samples that were then used in this study.

#### Mass Spec proteomics (excerpted from project 190 study documents)

Proteins were extracted from neat urine samples and digested into peptides by using the MStern blotting sample preparation protocol. To determine urinary proteome profiles, purified peptides were loaded on Evosep Evotips, separated via an online-coupled Evosep One HPLC and analyzed on a Bruker timsTOF Pro mass spectrometer with a data-independent acquisition method and a gradient length of 44 min. Analysis of raw spectra was performed with DIA-NN.

### Olink CSF and plasma proteomics

Plasma and CSF samples in the Stanford-5x and PPMI cohorts were sent to Olink Proteomics AB (Uppsala, Sweden. http://www.olink.com) for the quantification of up to 1536 proteins using a multiplex proximity extension assay [1]. This technology has been extensively vetted in biomarker studies and detailed methodology of the assay has been previously published [1]. Briefly, the proximity extension assay uses DNA oligonucleotide-labeled polyclonal antibodies which bind to each protein target. When two antibodies targeting different epitopes bind the same protein target, a proximity-dependent DNA ligation and elongation reaction can occur. The requirement for coincident binding leads to high specificity. The target protein levels can then be read out using quantitative PCR (qPCR) or next generation sequencing. This technology enables multiplex measurement of up to 96 or up to 384 protein targets in a single assay, depending on assay version. In the Stanford-5x cohorts, proteins from 13 different 96-protein panels were measured, resulting in quantification of 1196 proteins in both CSF and plasma samples. CSF and plasma protein levels were analyzed using the Cardiometabolic (v.3602), Cardiovascular II (v.5005), Cardiovascular III (v. 6112), Cell Regulation (v.3701), Development (v.3512), Immune Response (v.3201), Inflammation (v.3012), Metabolism (v.3402), Neuro Exploratory (v.3901), Neurology (v.8011), Oncology II (v.7002), Oncology III (v.4001) and Organ Damage (v.3301) 96-plex immunoassay Olink panels. In the PPMI cohort, four 384-protein panels from the Olink explore 1536 platform, the Cardiometabolic 384, Neurology 384, Oncology 384, and Inflammation 384, were run. Details on the exact assay version were not provided in study docs.

#### Olink data processing and quality control

A detailed description of the Olink data normalization and QC process can be found at https://olink.com/application/data-normalization-and-standardization/. Exact processing differs depending on assay version (qPCR-based readout or NGS-based readout).

In Stanford-5x, a qPCR-based readout was used. As previously described [1], eight control samples are run on each plate: two are external pooled plasma samples, which are used to assess potential intra-plate/run variation, three are Inter-Plate Controls (IPCs) and three are buffer blanks. The IPCs are formed from a pool of 92 antibodies. The median of the IPCs is used to normalize each assay and compensate for potential variation between runs and plates.

Briefly, protein expression data are reported in Normalized Protein eXpression (NPX), which is a normalized unit on a log_2_-scale. Calculation of NPX differs based on assay version, depending on if a qPCR readout or next generation sequencing readout was used. In Stanford-5x cohorts, a qPCR readout was used, and the NPX values are derived from the Ct or “threshold cycle”. This is the number of qPCR cycles needed for the signal to pass a fluorescence signal threshold. NPX is calculated from the Ct values using the following equations:

$${\text{Extension control}}:{\text{ CtAnalyte-CtExtension Control}}\, = \,{\text{dCtAnalyte}},$$

$${\text{Inter}} - {\text{plate control}}:{\text{ dCtAnalyte-dCtInter}} - {\text{plate Control}}\, = \,{\text{ddCtAnalyte}},$$

$${\text{Adjustment against a correction factor}}:{\text{ correction factor-ddCtAnalyte}}\, = \,{\text{NPXAnalyte}}.$$

In the PPMI cohort, an NGS readout was used. Detailed information on normalization and the calculation of NPX from NGS reads can be found in the PPMI project 196 study documents and on the Olink website.

### SomaScan proteomics

#### SomaScan assay

The SomaLogic SomaScan assay, which uses slow off-rate modified DNA aptamers (SOMAmers) to bind target proteins with high specificity, was used to quantify the relative concentration of 4979 protein targets in CSF and 7288 protein targets in plasma samples from Stanford-5x. The assay has been used in hundreds of studies and described in detail previously [15, 35]. Two versions of the SomaScan assay were used in this study. The v4 assay (4,979 protein targets) was used in CSF samples from Stanford-5x, and the v4.1 assay (7,288 protein targets) was used in plasma samples from Stanford-5x. All v4 probes are included in the v4.1 assay. Since protein levels across assay versions were not directly compared to each other, no bridging procedure was needed in the current study to harmonize values.

#### SomaScan normalization and QC

Standard Somalogic normalization, calibration, and quality control were performed on all samples [59–61, 69] by SomaLogic Inc. Detailed documentation can be found at https://somalogic.com/tech-notes/. Briefly, pooled reference standards and buffer standards are included on each plate to control for batch effects during assay quantification. Samples are normalized within and across plates using median signal intensities in reference standards to control for both within-plate and across-plate technical variation. Samples are further normalized to a pooled reference using an adaptive maximum likelihood procedure. Samples are additionally flagged by SomaLogic if signal intensities deviated significantly from the expected range and these samples were excluded from analysis. The resulting expression values are the provided data from Somalogic and are considered “raw” data. Raw data was then log10 transformed to reduce heteroscedasticity and increase power in downstream statistical modeling.

### Statistical analyses

To examine demographic and clinical group differences, we used a non-parametric Wilcoxon sign-rank test or a non-parametric one-way ANOVA on ranks (Kruskal Wallis *H*-test).

When using longitudinal sample data, we ran differential expression analysis on protein levels using a multi-level linear-mixed effects model controlling for age, sex, race, ethnicity, and sample-relatedness. Sample-relatedness refers to longitudinally collected samples from a single individual, which we expect to be more correlated than samples from different individuals. When looking at samples from one timepoint only, we used a linear model controlling for age, sex, race, and ethnicity.

We used Benjamini–Hochberg false discovery rate control across the number of detected proteins to account for multiple testing. We studied the association between DDC levels and clinical measures of disease severity (MDS-UPDRS III, LEDD, MoCA) using linear regression analyses corrected for age and sex. We used principal component analysis to explore the relationship between global differences in protein expression profile and clinical/demographic variables. We tested correlations between principal components with a spearman correlation test with Bonferroni correction for multiple testing. All statistical analysis was done in R 4.0.3. We used the package lmerTest [19] and the dream function from the R package variancePartition [17] for mixed effects models. We used the glm function with a binomial link in R 4.0.3 to perform binary logistic regression. We used the pROC [46] and multiROC [68] packages in R 4.0.3 to generate and visualize receiver operator sensitivity–specificity curves and calculate area under the ROC curve.

## Results

We utilized five independent Stanford research cohorts (Stanford-5x) and two independent cohorts from the Parkinson’s Progression Marker Initiative (PPMI) to identify biomarkers in this study (Fig. 1, Supplementary Table 1). We began by analyzing blood and CSF proteomics from Stanford-5x using two methods: proximity extension assay (Olink) and aptamer precipitation assay (SomaScan). We then replicated results in PPMI 1 using Olink and expanded the analysis into urine with LC–MS/MS proteomics from PPMI 2.

### CSF and plasma proteomics identifies biomarkers of PD

We first compared the Olink CSF and plasma proteomes of people with PD, HC, and AD-s in our discovery cohorts (Stanford-5x). The CSF study population included 71 PD, 78 HC, and 52 AD-s samples. The plasma study population included 68 PD, 105 HC, and 77 AD-s samples. Analysis of the first 10 principal components for CSF and plasma proteins indicated that there was not a statistically significant global difference in protein expression between the disease groups. The first principal component in CSF did show a significant correlation with age (spearman *p* = 0.0005) (Supp Fig. 1).

**Fig. 2:**
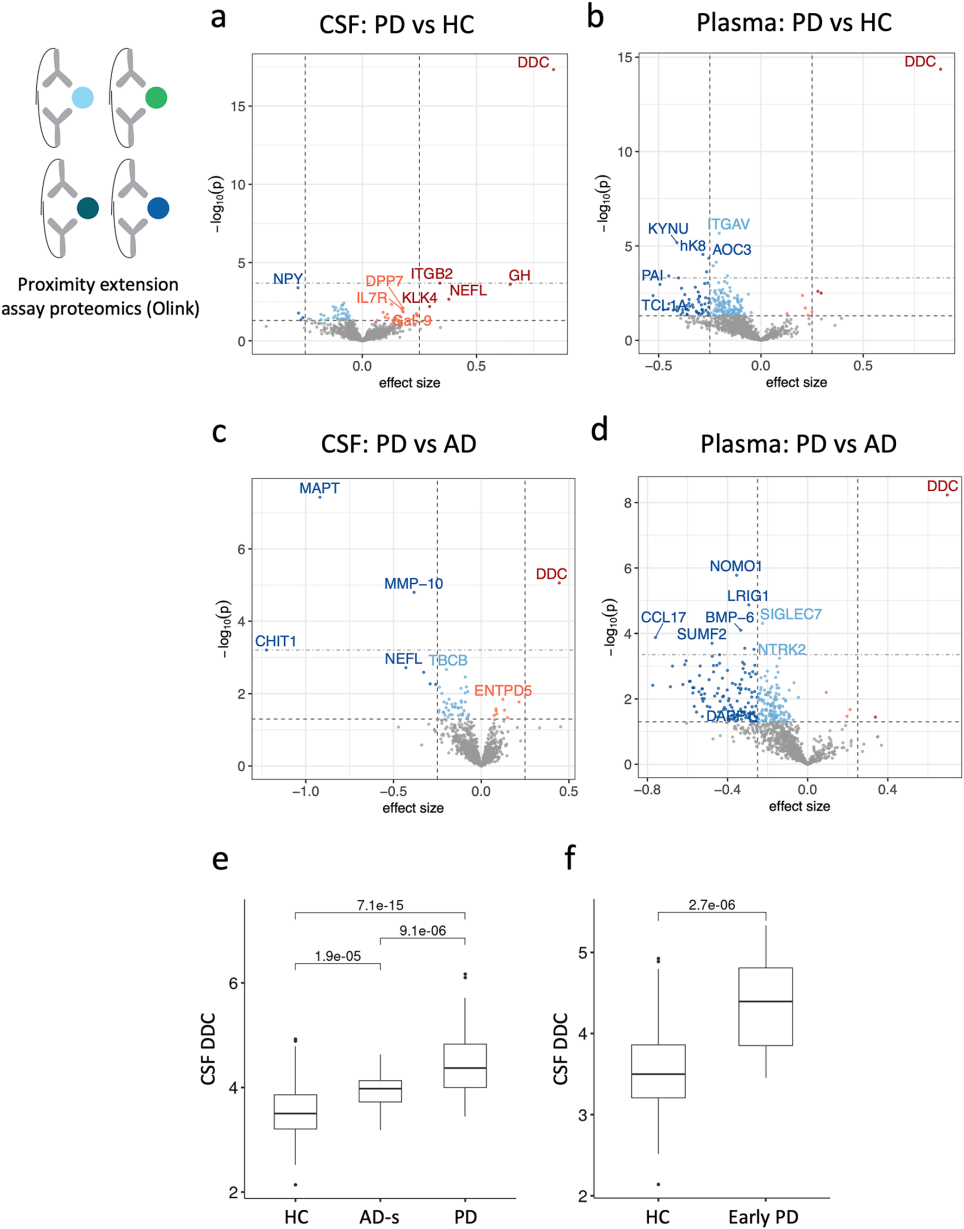
Differential expression analysis of CSF and plasma from Stanford-5x using Olink proximity extension assay proteomics identifies DDC (DDC) as a top hit in both tissues. Top horizontal dotted line indicates FDR significance threshold, bottom horizontal dotted line indicates raw p-value threshold. Dotted vertical lines indicate an arbitrary 0.25 effect size cutoff for moderate-effect proteins. Significant hits are shaded by effect size cutoff. **a** Results comparing Parkinson’s disease participants to healthy controls in CSF. **b** Results comparing Parkinson’s disease participants to Alzheimer’s disease participants in CSF. **c** Results comparing Parkinson’s disease participants to healthy controls in plasma **d** Results comparing Parkinson’s disease participants to Alzheimer’s disease participants in plasma. **e** CSF DDC levels plotted per disease group. **f** Plasma DDC levels plotted per disease group. Statistics from non-parametric one-way ANOVA test

To identify proteins whose expression differed between disease groups, we performed differential protein expression analysis on the Olink CSF and plasma proteomes using linear mixed-effects models while controlling for age, sex, education, ethnicity, and repeated longitudinal sampling (Fig. 2). When comparing people with PD to HC, there was 1 significant hit in CSF (Fig. 2a, Supplementary Table 2) and 10 significant hits in plasma (Fig. 2b, Supplementary Table 3) after proteome-wide multiple testing correction. Comparing people with PD to AD-s, there were three significant hits in CSF (Fig. 2c, Supplementary Table 4) and 9 significant hits in plasma (Fig. 2d, Supplementary Table 5). We also performed differential expression for age and sex (Supp Fig. 2), and replicated known top hits such as PTN and WFDC2 as significantly up in aging in plasma [21, 67], and CGA, CGB3, and PSPN as significantly differential between sexes [21, 64].

The protein DOPA decarboxylase (DDC), also known as aromatic l-amino acid decarboxylase (AADC EC 4.1.1.28), was the top upregulated hit in PD when compared to both HC and AD-s, in both CSF and plasma (Fig. 2e). DDC was most elevated in PD patients, but it was also significantly upregulated in AD-s relative to HC. DDC remained significantly elevated after controlling for age, sex and levodopa equivalent daily dose [LEDD (*F*_2,200_ = 10.515, *p* < 0.001, partial *η*^2^ = 0.095]. We also looked specifically at individuals in our study with Early PD (≤ 2 years since diagnosis) compared to HC, and found significantly elevated concentrations in Early PD, *F*_113_ = 9.101*, p* = 2.7e−6 (Fig. 2f).

We used GTEx [7] to look at DDC expression levels in different tissues and verified that in the brain, DDC is highly expressed in the substantia nigra (Supp Fig. 3). DDC is directly involved in monoamine synthesis, most notably dopamine synthesis in dopaminergic neurons, though it plays a role in synthesis of serotonin and other trace amines as well. This mechanistic link to known PD pathogenesis makes it an appealing biomarker candidate for PD.

**Fig. 3:**
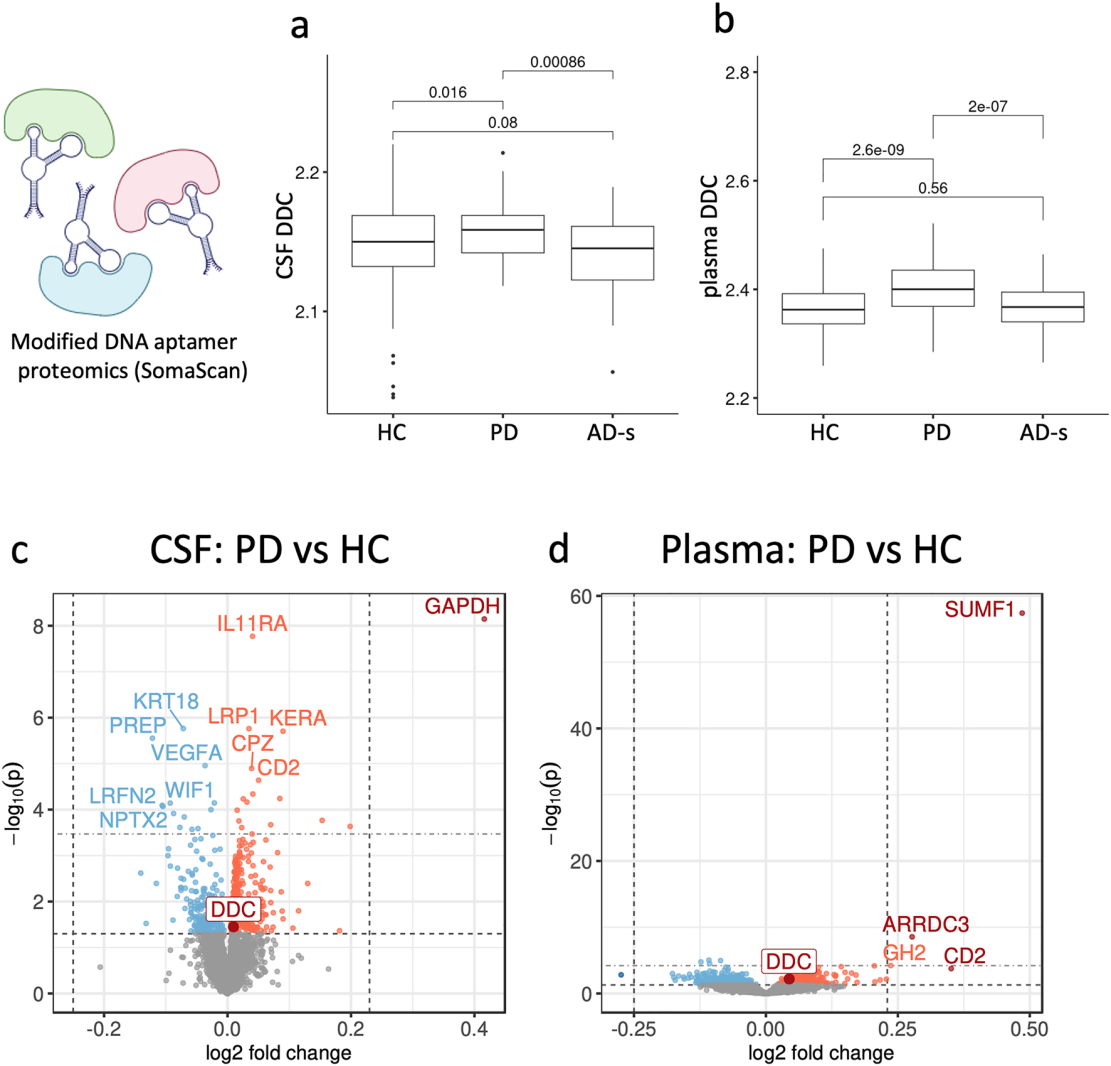
Differential expression analysis of CSF and plasma from Stanford-5x using SomaScan modified DNA aptamer proteomics replicates DDC as a hit and identifies additional top hits GAPDH and SUMF1. **a** DDC levels in CSF measured by SomaScan aptamer. **b** DDC levels in plasma measured by SomaScan aptamer. **c, d** Proteome-wide differential expression analysis. Top horizontal dotted line indicates FDR significance threshold, bottom horizontal dotted line indicates raw p-value threshold. Dotted vertical lines indicate an arbitrary 0.25 effect size cutoff for moderate-effect proteins. Significant hits are shaded by effect size cutoff. DDC is highlighted for comparison to Fig. 1. **c** Parkinson’s disease participants vs healthy participants in CSF. **d** Parkinson’s disease participants vs healthy participants in plasma. Statistics in A and B from non-parametric one-way ANOVA test

To expand our findings, we also analyzed 385 CSF samples (71 PD, 253 HC, 61 AD-s) and 1164 plasma samples (249 PD, 652 HC, 263 AD-s) using an orthogonal proteomics platform, the SomaScan assay, in Stanford-5x (Fig. 3). Unlike Olink, which utilizes a polyclonal antibody pool against each target, SomaScan uses a single high affinity aptamer (raised in-vitro) to bind each protein target. For this reason, Olink and SomaScan commonly have differential sensitivity to proteoform changes such as post-translational modification or degradation. We found that DDC was also upregulated in both CSF and plasma using the SomaScan assay (Fig. 3a, b), but the effects were smaller in magnitude than in the Olink assay. Interestingly, we did not find DDC was upregulated in AD-s participants using SomaScan, despite having more statistical power. These difference across assays may suggest that there are different DDC proteoforms in AD-s and PD, which could be investigated in future research to identify even more specific DDC-based biomarkers of PD, similar to phosphorylated Tau species in AD-s.

In a proteome-wide analysis of the SomaScan data, there were 29 significant hits in CSF PD vs HC and 7 significant hits in plasma PD vs HC after FDR correction (Fig. 3c, d, Supplementary Tables 6–7). GAPDH in CSF and SUMF1 in plasma stand out as significant proteins with large effect sizes. SUMF1 encodes sulfatase-modifying factor 1 (aka formylglycine-generating enzyme, FGE), which is responsible for generating the active site of all known sulfatases in the body [8, 50]. Although it has not been previously linked to PD, mutations in SUMF1 and its sulfatase targets cause a diversity of rare lysosomal storage diseases, many of which have large phenotypic overlap with PD [45, 53]. For example, mutations in the lysosomal storage disorder-causing gene GBA are a common risk factor for PD [53].

**Fig. 4:**
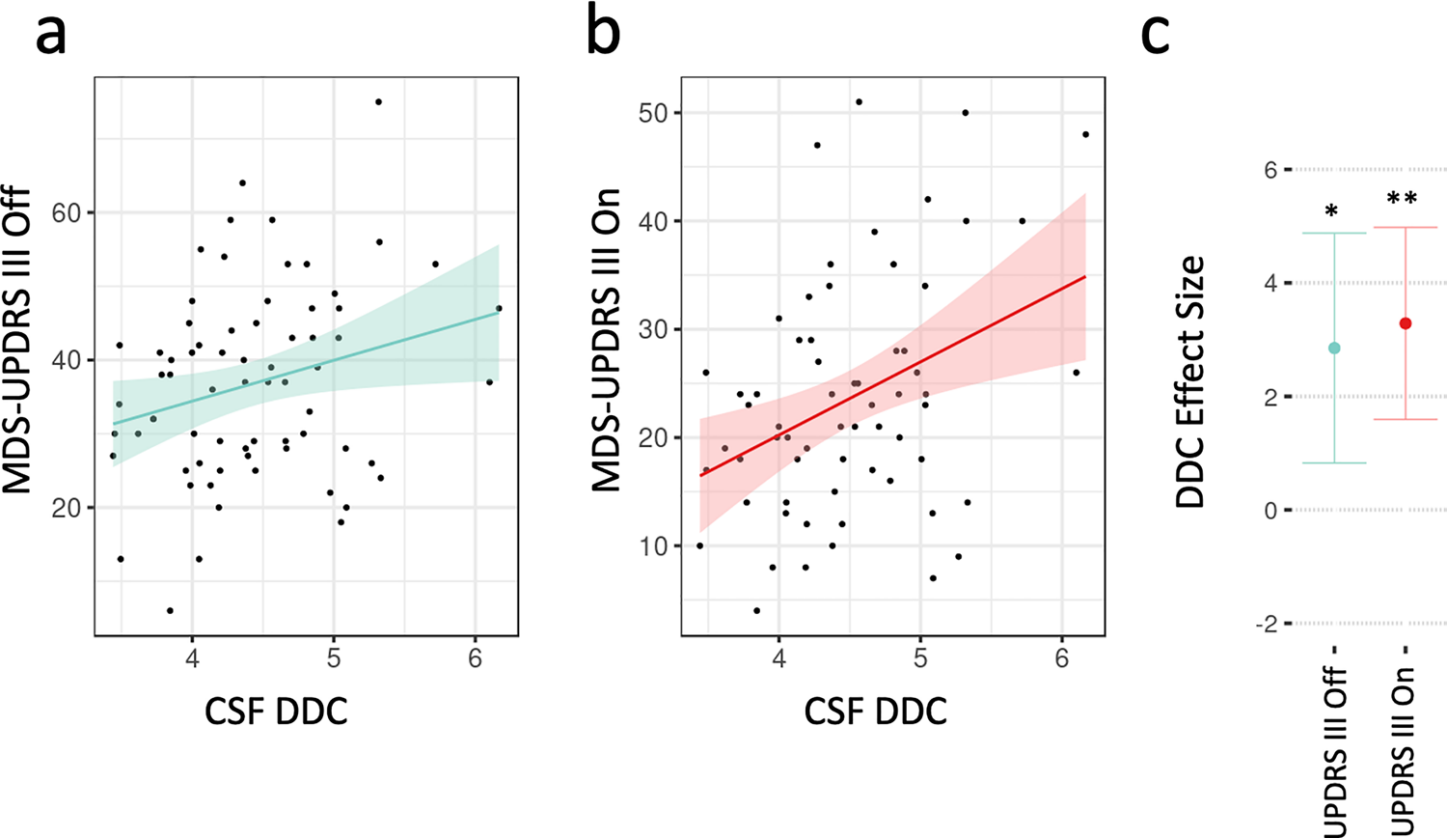
CSF DDC levels are correlated with motor function in PD participants. **a** Correlation between MDS-UPDRS III Off score and CSF DDC level. **b** Correlation between MDS-UPDRS III On score and CSF DDC level. **c** Effect size estimate and *p*-value of relationship between CSF DDC and UPDRS III scores after controlling for age and sex effects on scores. 95% confidence intervals are shown. MDS-UPDRS III Off *p* = 0.022. MDS-UPDRS III On *p* = 0.0017

Averaged across CSF and plasma in both platforms, DDC was the most significant hit (*p* = 0.01), and given its plausible role in PD, we next tested if ADDC levels in CSF and plasma were associated with PD symptom severity and could thus have potential prognostic and clinical utility. We focused on the Olink assay for this analysis because of the larger effect size. We found that DDC levels in CSF were significantly associated with severity of motor symptoms assessed on the MDS-UPDRS III Off (*β* = 2.85, *p* = 0.022) (Fig. 4a), and On scores (*β* = 3.29, *p* = 0.0017) (Fig. 4a–c).

### DDC is elevated in prodromal PD and is associated with disease symptom severity

DDC’s direct involvement in dopamine synthesis raises questions about the role of dopaminergic medications in this finding. Although our analysis in Stanford-5x suggested that LEDD did not explain the relationship between DDC levels and symptom severity, the possibility that elevated DDC levels are driven by dopamine replacement therapy could not be fully excluded in Stanford-5x because nearly all PD participants were taking some form of dopamine replacement therapy at the time of blood and CSF collection.

To rule out this possibility, we turned to an unpublished Olink CSF and plasma proteomics study done in untreated, newly diagnosed PD participants (de novo PD) in the Parkinson’s Progression Marker Initiative (PPMI), a multicenter international prospective cohort study [28, 29]. Participants in the PPMI 1 de novo cohort are not taking any medications for PD at their baseline visit, so we first restricted analysis to baseline samples to rule out any medication influences. There were 243 baseline CSF samples (69 PD, 130 HC, 44 prodromal) and 262 baseline plasma samples (74 PD, 130 HC, 58 prodromal).

**Fig. 5:**
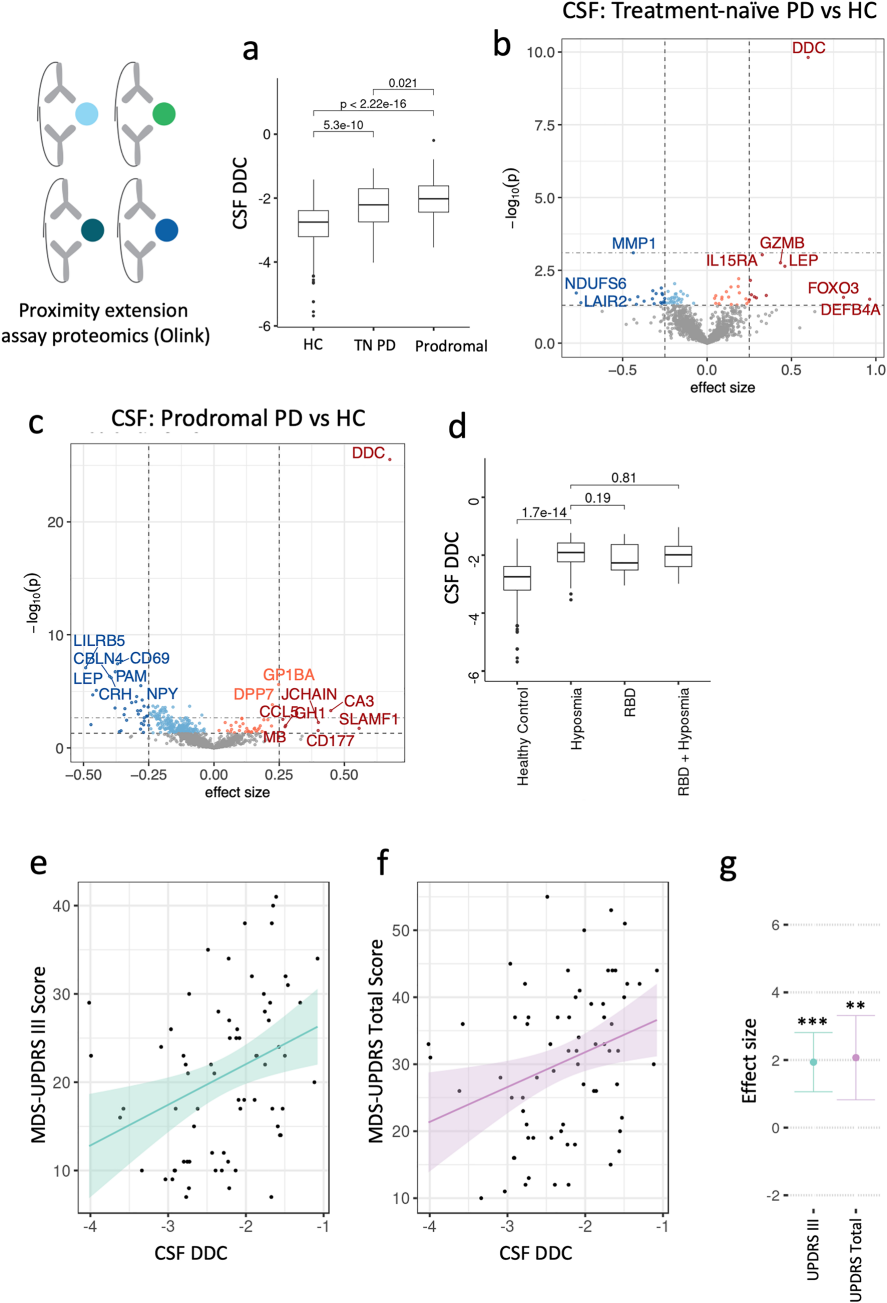
Replication of DDC findings in CSF Olink proteomics from treatment-naïve and prodromal PD participants in the PPMI 1 cohort. **a** CSF DDC is significantly elevated in treatment-naïve PD (TN PD) and prodromal PD. **b, c** Proteome-wide differential expression analysis of TN PD vs healthy controls (B) and prodromal PD vs healthy controls (C) identifies DDC as the most differentially expressed protein. Top horizontal dotted line indicates FDR significance threshold, bottom horizontal dotted line indicates raw *p*-value threshold. Dotted vertical lines indicate an arbitrary 0.25 effect size cutoff for moderate-effect proteins. Significant hits are shaded by effect size cutoff. **d** CSF DDC is elevated in both hyposmic and RBD prodromal subtypes. Hyposmic participants trend higher but the effect is not significant (*p* = 0.19). **e** Correlation between MDS-UPDRS III score and CSF DDC level in treatment-naïve, baseline PD participants in PPMI. **f** Correlation between MDS-UPDRS total score and CSF DDC level in treatment-naïve, baseline PD participants in PPMI. **g** Effect size estimate and p value of relationship between CSF DDC and MDS-UPDRS III scores after controlling for age and sex effects on scores. 95% confidence intervals are shown. MDS-UPDRS III *p* = 0.00018, MDS-UPDRS total score *p* = 0.0063

In treatment naïve, newly diagnosed, baseline PD participants, CSF DDC was significantly elevated compared to HC (Fig. 5b). In a proteome-wide analysis, DDC was the most significantly upregulated protein in CSF, and the only protein which passed the proteome-wide FDR significance threshold. (Fig. 5c, supplementary Table 8). DDC was even more strongly upregulated in prodromal participants with DAT deficit and RBD or hyposmia, where it remained the most significantly upregulated protein (Fig. 5d). This suggests that changes in DDC may happen early in the disease course before motor symptoms emerge. In prodromal participants, there were an additional 3 upregulated proteins and 56 downregulated proteins which passed proteome-wide FDR correction (Supplementary Table 9). There was no significant difference in DDC levels between prodromal participants with hyposmia and those with RBD, though those with hyposmia were trending higher (*p* = 0.19, Fig. 5e). CSF DDC was also significantly elevated in non-manifesting LRRK2 and GBA mutation carriers, though sample sizes were too small to draw a robust conclusion on this point (extended data Fig. 4).

Given these findings, we tested if DDC levels increase over time in longitudinally sampled PD or prodromal participants using a linear mixed effects model. This was difficult to resolve In PD participants, since after the baseline visit most participants begin dopaminergic medications to manage their PD. We found that DDC levels did increase significantly over time in PD participants, but this was confounded with LEDD. In prodromal participants, there was a trend towards increased DDC levels over time (*B* = 0.04, *p* = 0.07) but the effect size was small and only a small number of prodromal participants had multiple samples. Larger studies are needed to understand the relevance of this increase in prodromal PD.

Plasma DDC levels were not significantly elevated in de-novo PD participants at baseline or in prodromal PD participants (Extended data Fig. 5, Supplementary Table 10–11). This is in contrast to the findings in Stanford-5x, suggesting that medications may have an impact on DDC plasma levels. In both PD vs HC and Prodromal vs HC comparisons, no proteins in plasma passed proteome-wide FDR correction.

In PPMI 1, we replicated the finding that CSF DDC levels are significantly associated with symptom severity. In baseline samples from treatment-naïve PD participants, CSF DDC levels were significantly associated with MDS-UPDRS III score (*β* = 1.94, *p* = 0.0003) and MDS-UPDRS total symptom score (*β* = 2.07, *p* = 0.007) (Fig. 5F–H).

In contrast to these findings on DDC, CSF alpha-synuclein level was not significantly associated with any motor or nonmotor symptoms in any participant subset (Supp Fig. 6), suggesting that DDC levels could be an earlier and more quantitative correlate of clinical symptoms than CSF alpha-synuclein.

### DDC and other proteins are elevated in PD urine samples

When considering the utility of clinical biomarkers for diagnostics and disease monitoring, an important feature is the invasiveness and ease of sample collection. Urine is an ideal biofluid for disease biomarkers because collection is simple and completely non-invasive. Therefore, to fully understand protein biomarkers of PD and early PD, we next examined an unpublished LC–MS/MS proteomics study conducted in a separate group of participants from the PPMI cohort (PPMI 2) with a focus on carriers of GBA or LRRK2 mutations (Fig. 7a). This study included 1156 samples (549 PD, 140 HC, and 467 non-manifesting carriers with high risk GBA or LRRK2 mutations) from 983 participants. 6487 proteins were detected in the study, but we restricted the analysis to 4222 proteins which were detected in at least 50 samples from every group to avoid bias from sparsely detected outliers.

**Fig. 6:**
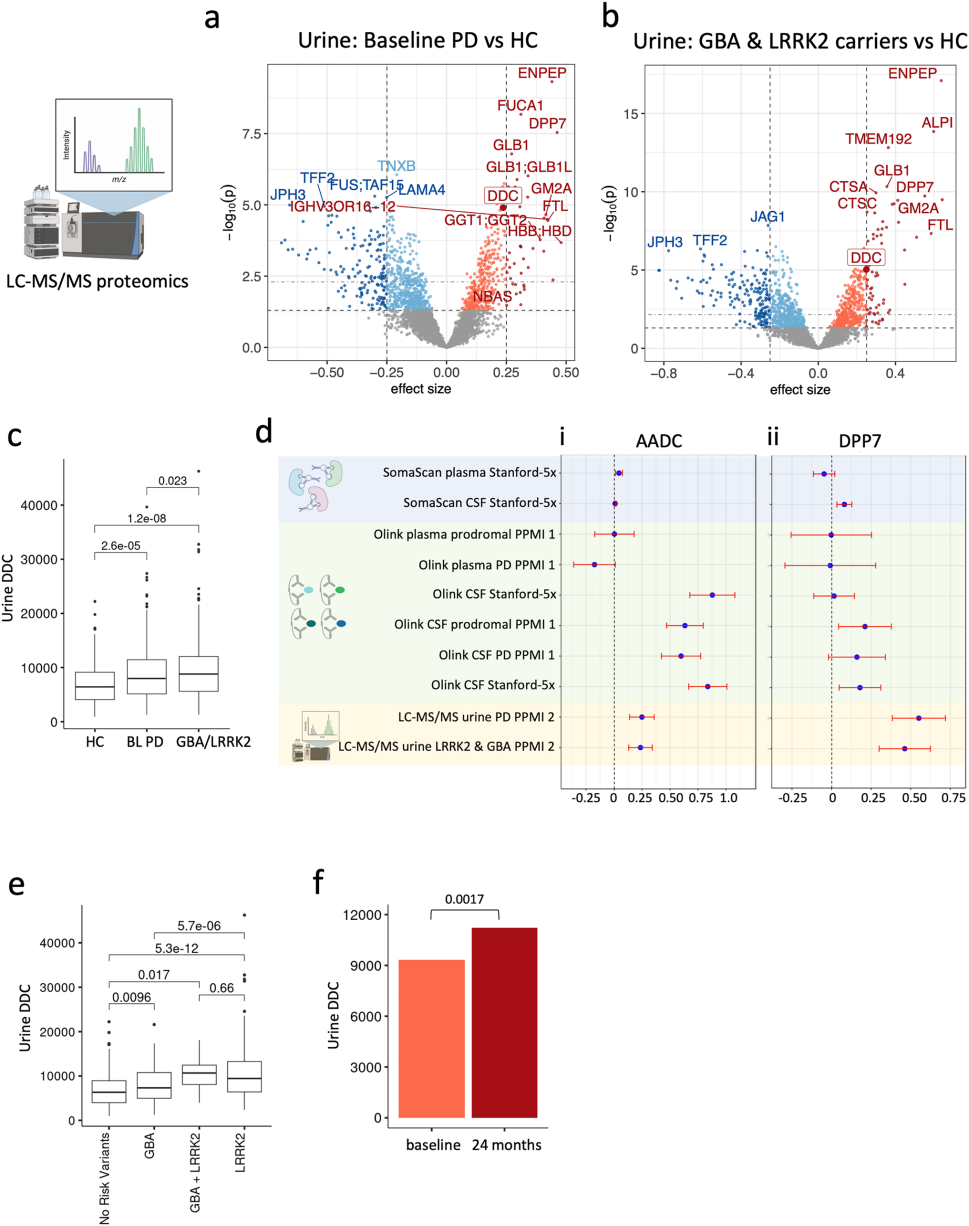
Liquid chromatography-mass spectrometry-mass spectrometry (LC–MS/MS) proteomics of urine samples in the PPMI 2 cohort. **a, b** Proteome-wide differential expression analysis of LC–MS/MS urine data in baseline PD vs healthy controls (**A**) and non-manifesting GBA and LRRK2 carriers vs healthy controls (**B**) identifies multiple strong hits including DDC. Top horizontal dotted line indicates FDR significance threshold, bottom horizontal dotted line indicates raw p-value threshold. Dotted vertical lines indicate an arbitrary 0.25 effect size cutoff for moderate-effect proteins. Significant hits are shaded by effect size cutoff. **c** Urine DDC levels are elevated in baseline PD (BL PD) and non-manifesting GBA and LRRK2 carriers. **d** Summary of differential expression effect sizes across all cohorts and measurement technologies for DDC (i) and DPP7 (ii). Error bars represent 95% confidence intervals. DDC is significantly elevated in all tissues by all measurement technologies, though effect strength varies across technologies and tissues. DDP7 is elevated in CSF and urine across all 3 technologies, with strongest effects in urine. **e** Urine DDC levels in non-manifesting carrier types. DDC is significantly elevated in both LRRK2 and GBA carriers, and is significantly more elevated in LRRK2 carriers. **f** Urine DDC levels go up by ~ 10% in LRRK2 carriers with repeated sampling at baseline and 24 months. Statistic is *p*-value of time covariate in a linear mixed-effects model which accounts for individual differences in baseline DDC levels, as well as age and sex. Bars are mean DDC levels at each timepoint in males of mean study population age

We performed differential expression analysis in baseline PD samples (472 PD and 140 HC samples) and in non-manifesting carrier participant samples (140 HC, 363 non-manifesting carriers). In PD vs HC analysis, 254 proteins were significantly downregulated, and 165 proteins were significantly upregulated after proteome-wide FDR adjustment (Fig. 6b, Supplementary Table 12). We discovered, however, that due to a difference in recruitment for genetic carriers in PPMI 2, some PD baseline participants in this study were not treatment-naïve. We repeated differential expression analysis using only treatment-naïve baseline PD samples (298 de novo PD, 140 HC), and found that effects between the whole baseline population and the treatment-naïve only population correlated extremely well (*r* = 0.91, *p* < 2.2e−16) (Supplementary Fig. 7, Supplementary Table 13). This suggests the main effect of this difference was reduced statistical power in the treatment-naïve group due to smaller sample size. No proteins in the treatment-naïve only analysis passed proteome-wide significance, but top hits including DDC (effect size = 0.15, *p* = 0.014) remained the most statistically significant hits.

In PD vs non-manifesting carrier analysis, 329 proteins were significantly downregulated, and 259 proteins were significantly upregulated after proteome-wide FDR adjustment (Fig. 6c, Supplementary Table 14). Multiple top hits, including ENPEP, WFDC2, JPH3, GLB1, DPP7, and DDC had consistent and large effect sizes across baseline PD, de novo PD, and non-manifesting carrier groups.

DDC was a top upregulated protein in both baseline de novo PD and non-manifesting carrier urine samples (Fig. 6B–E, Supp Fig. 7), validating this biomarker across three different biofluids and three orthogonal proteomics platforms. Another notable large effect-size hit which was validated across all 3 proteomics platforms in CSF and urine in both baseline de novo PD and non-manifesting carriers was Dipeptidyl Peptidase 7 (DPP7), also known as Dipeptidyl Peptidase 2 (DPP2), a serine protease of unknown function. This protein was most significantly upregulated in urine of PD and non-manifesting carrier participants, where its effect was larger than DDC (Fig. 6e, f). DPP7 has a known role in immune cell quiescence, and increased DPP7 enzymatic activity has been repeatedly observed in the CSF of PD patients [25, 26]. Related DPP proteins have roles in synapse formation and maintenance, gut inflammation, and immune activation [20, 27, 41], suggesting that DPP7 may also play a role in these processes which have been implicated in PD pathogenesis.

The urine study allowed us to assess the relationship between DDC and genetic risk factors of PD. DDC was significantly elevated in non-manifesting GBA and LRRK2 mutation carriers compared to healthy participants without genetic risks (Fig. 6g). DDC was also significantly elevated in LRRK2 carriers compared to GBA carriers, but the expression between LRRK2 carriers and dual GBA/LRRK2 carriers was indistinguishable. Repeated sampling at baseline and a 2-year follow-up visit in 98 non-manifesting LRRK2 carriers allowed us to assess if urine DDC levels increased over time using a linear mixed effects model. Urine DDC levels increased significantly in LRRK2 carriers (*B* = 950, *p* = 0.0022) by an average of 10% over this time (Fig. 6h).

### DDC discriminates PD and prodromal PD from AD and HC

**Fig. 7:**
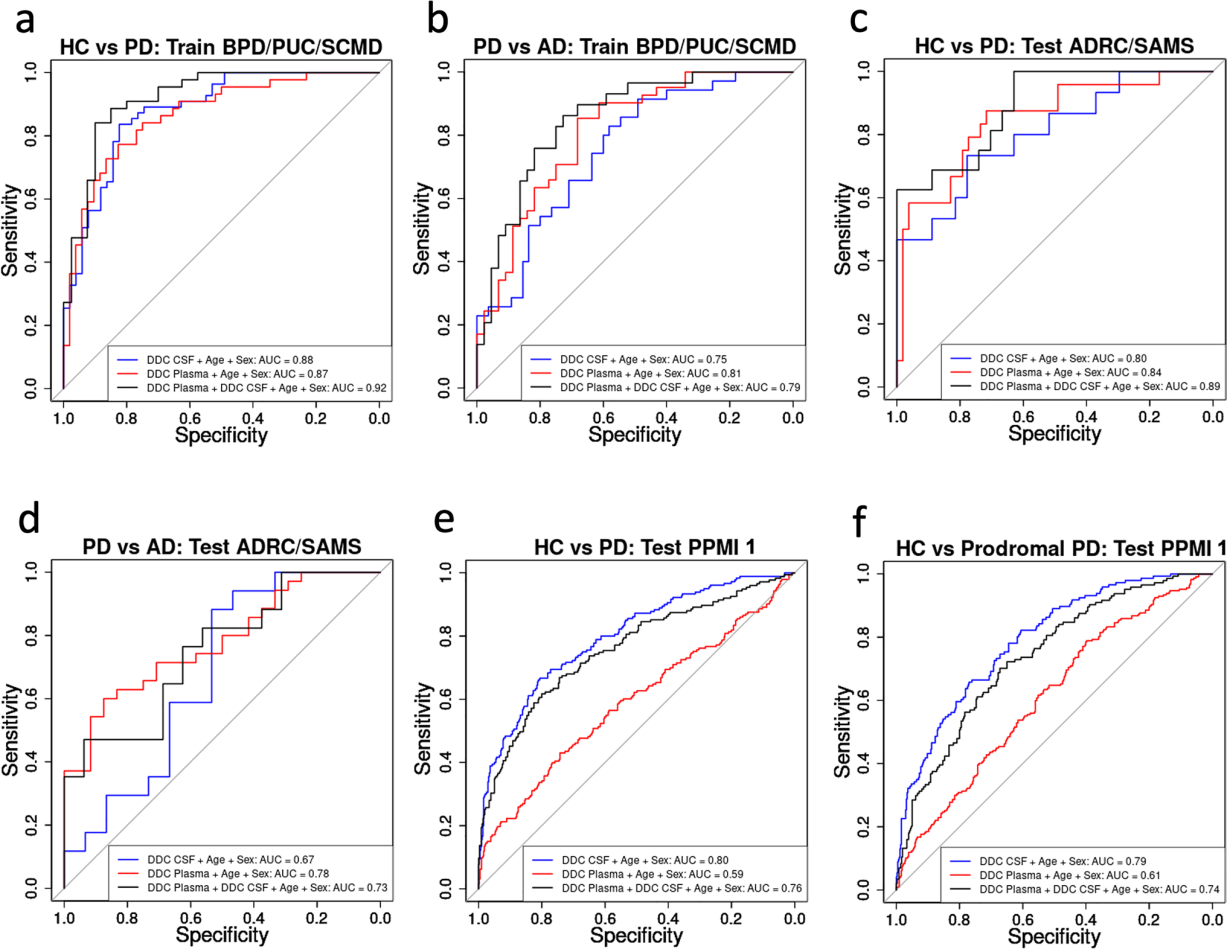
Development and testing of logistic regression classifiers to discriminate PD from HC and AD-s participants using DDC levels in CSF and plasma. Receiver operating characteristic (ROC) sensitivity–specificity curves of classifier performance are plotted. Area Under the Curve (AUC) is a measure of classifier performance, with an AUC of 1 being perfect classification with no false positives (perfect specificity) and no false negatives (perfect sensitivity). The diagonal (AUC = 0.5) represents a random guess. Classifier using CSF DDC levels is shown in blue. Classifier using plasma DDC levels is shown in red. Classifier using both CSF and plasma DDC levels shown in black. All classifiers include age and sex as additional covariates. **a** Performance on PD vs HC in the Stanford-5x sub-cohorts BPD, PUC, and SCMD, which were used to train the model. CSF AUC = 0.88. Plasma AUC = 0.87. Combined AUC = 0.92. **b** Performance on PD vs AD-s in the Stanford-5x sub-cohorts BPD, PUC, and SCMD. CSF AUC = 0.75. Plasma AUC = 0.81. Combined AUC = 0.79. **c** Performance on PD vs HC in the held-out test sub-cohorts from Stanford-5x ADRC and SAMS. CSF AUC = 0.80. Plasma AUC = 0.84. Combined AUC = 0.89. **d** Performance on PD vs AD-s in the held-out test sub-cohorts from Stanford-5x ADRC and SAMS. CSF AUC = 0.67. Plasma AUC = 0.78. Combined AUC = 0.73. **e** Performance on PD vs HC in the completely independent test cohort PPMI 1. CSF AUC = 0.80. Plasma AUC = 0.59. Combined AUC = 0.76. **f** Performance on prodromal PD vs HC in the completely independent test cohort PPMI 1. CSF AUC = 0.79. Plasma AUC = 0.61. Combined AUC = 0.74

Given that CSF DDC levels are consistently elevated in people with PD compared to HC and AD-s, we tested if DDC levels could accurately discriminate PD from HC and AD-s participants. We trained a logistic regression classifier of PD diagnosis against HC and AD-s diagnosis within a subset of the Stanford-5x cohorts (PUC/BPD/SCMD), and evaluated its performance in the remaining held-out cohorts (ADRC/SAMS) and on the independent PPMI 1 CSF and plasma datasets (Fig. 7a–e). We found that both CSF and plasma DDC levels were capable of discriminating PD in Stanford-5x with promising sensitivity and specificity (CSF ROC AUC = 0.88, plasma ROC AUC = 0.87), but CSF DDC outperformed plasma DDC in the larger fully independent test cohort PPMI (CSF ROC AUC = 0.80, plasma ROC AUC = 0.59). This makes sense given that plasma DDC levels were found to be medication-dependent while CSF DDC levels were not—classifiers trained on CSF DDC levels from patients taking dopaminergic medications in Stanford-5x were still able to diagnose PD in de novo PPMI 1 participants. We also applied the classifier to prodromal participants in PPMI 1 (Fig. 7f) and found CSF DDC levels did equivalently well at diagnosing prodromal PD (CSF ROC AUC = 0.79) without any adjustment of the algorithm. These data suggest CSF DDC may hold promise as a diagnostic biomarker of early PD even before the emergence of motor symptoms.

## Discussion

In the search for new biomarkers of Parkinson's disease (PD), we analyzed proteomics data from multiple large human studies of PD, AD, and prodromal PD, totaling approximately 5000 samples across three orthogonal proteomics platforms in CSF, plasma, and urine. We discovered multiple promising candidate biomarkers in each biofluid, including the proteins DDC, DPP7/2, SUMF1, ENPEP, WFDC2, and others. While an in-depth investigation of all the hits uncovered is beyond the scope of this work, this study serves as a powerful resource for the field to investigate candidate genes of interest in multiple tissues and demonstrates the utility of proteomics in biomarker discovery.

This study highlights the discovery of DDC as a multi-tissue biomarker candidate for both early diagnosis and disease monitoring of PD. We and others have recently suggested DDC as a biomarker of PD status in CSF based on findings from Olink proteomics [4, 38, 39, 49]. The findings reported here are a comprehensive and unbiased analysis of DDC as a biomarker across CSF, plasma, and urine using three proteomics platforms and considering multiple stages of PD. Our findings indicate that DDC levels are consistently elevated in CSF and urine of PD patients, regardless of dopamine replacement therapy, including pre-motor disease stages and newly diagnosed, treatment-naïve individuals. We demonstrated that CSF DDC levels correlate with clinical symptom severity and can be used to identify treatment-naïve PD and prodromal PD. These data strongly suggest that DDC, previously overlooked due to its association with L-DOPA therapy, could be a specific marker for monoaminergic neuron degeneration in PD.

The discovery of DDC elevated in the urine of de-novo PD and genetic carriers is particularly significant, since the impact of a simple, scalable, and non-invasive diagnostic test for early PD would be substantial. This finding, as well as the discovery of other promising early PD biomarkers in urine including DPP7/2, ENPEP, WFDC2, JPH3, GLB1, and others, should be replicated in additional cohorts with multiple detection approaches to understand the full utility of urine protein testing in PD. The finding that DDC is most strongly elevated in LRRK2 carriers is also of particular interest. Existing αSyn-SAA tests are unlikely to be helpful in many LRRK2-PD or Parkin-PD patients, who lack Lewy body pathology despite substantial nigrostriatal degeneration [52]. A recent αSyn-SAA study in PPMI found that αSyn-SAA was unable to identify LRRK2 carriers [55].

This study raises crucial questions about whether the elevation of DDC in CSF and urine are a direct consequence of neuronal loss, a compensatory upregulation due to neurodegeneration, or some other mechanism. Previous studies have suggested that the increasing loss of endogenous DDC in the brain leads to waning treatment responses to dopaminergic medication over time [6, 42, 51]. However, the changes in DDC early in the disease and in genetic carriers demonstrated in this study are not understood. Currently, phase II clinical trials are investigating increasing DDC enzymatic expression via gene therapy as a potential therapeutic intervention for PD motor fluctuations [5, 12]. Our data raise the need for further study into the mechanisms of elevated DDC levels. If elevated CSF levels of DDC are due to compensatory upregulation of DDC elsewhere in the brain as a response to nigrostriatal degeneration, this could suggest that the location of DDC in the brain, or other non-dopamine functions of nigrostriatal neurons, play a key role in disease symptoms and progression.

CSF DDC also shows promise as a quantitative biomarker for monitoring disease progression and motor symptom severity in PD. This capability is particularly important given the limitations of current clinical measures such as the UPDRS or MDS-UPDRS, which are subjective and can be affected by large placebo effects [14, 22], a significant challenge for exam-based outcome measures in clinical trials. The ability of CSF DDC to reflect underlying disease severity has the potential to facilitate the development of new treatments and provide more objective endpoints for clinical trials.

In conclusion, our research reports the differentially regulated levels of hundreds of proteins in CSF, blood plasma, and urine in multiple tissues in prodromal PD and PD. We highlight the discovery of CSF and urine DDC as promising novel biomarkers, which may serve as valuable adjuncts for both monitoring and diagnosing PD.

### Supplementary Information

Below is the link to the electronic supplementary material.

Supplementary file1 (XLSX 3509 KB)

Supplementary file2 (PDF 1804 KB)

## Data Availability

Stanford-5x data used in this study will be made available on reasonable request to the Stanford-ADRC data release committee, https://web.stanford.edu/group/adrc/cgi-bin/web-proj/datareq.php. All Stanford-ADRC data will be made publicly available after an embargo period at this link: https://twc-stanford.shinyapps.io/adrc/. This analysis used data openly available from PPMI, see https://www.ppmi-info.org/ for more info.
