# Supplementary material for: Comprehensive proteomics of CSF, plasma, and urine identify DDC and other biomarkers of early Parkinson’s disease
Source: Acta Neuropathol. 2024 Mar 11;147(1):52. doi: 10.1007/s00401-024-02706-0 (PMC10927779; doi:10.1007/s00401-024-02706-0)
Supplement: Supplementary file 2 — Supplementary file2 (PDF 1804 KB) [file 401_2024_2706_MOESM2_ESM.pdf]

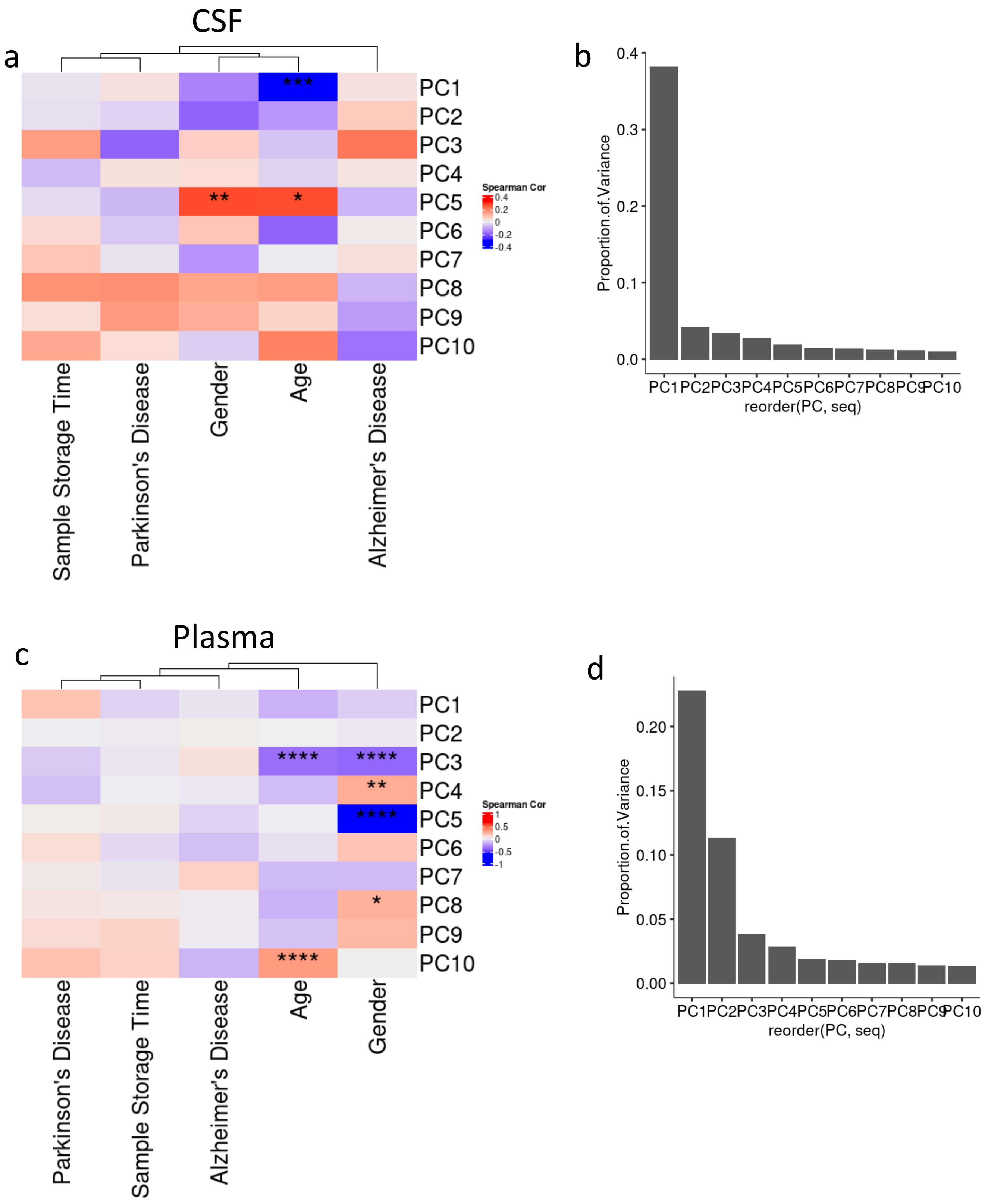

Supplementary Figure 1. Principal component analysis results for global protein expression in CSF and plasma. **a,c)** Correlation between top 10 principal components from CSF (A) or plasma (C) protein expression and demographic/disease diagnostic variables of interest. * indicates Bonferroni-adjusted p < 0.05. ** indicates Bonferroni-adjusted p < 0.01. **b,d)** Scree plots showing

the proportion of variance explained by each of the top 10 principal components in CSF (B) and plasma (D).

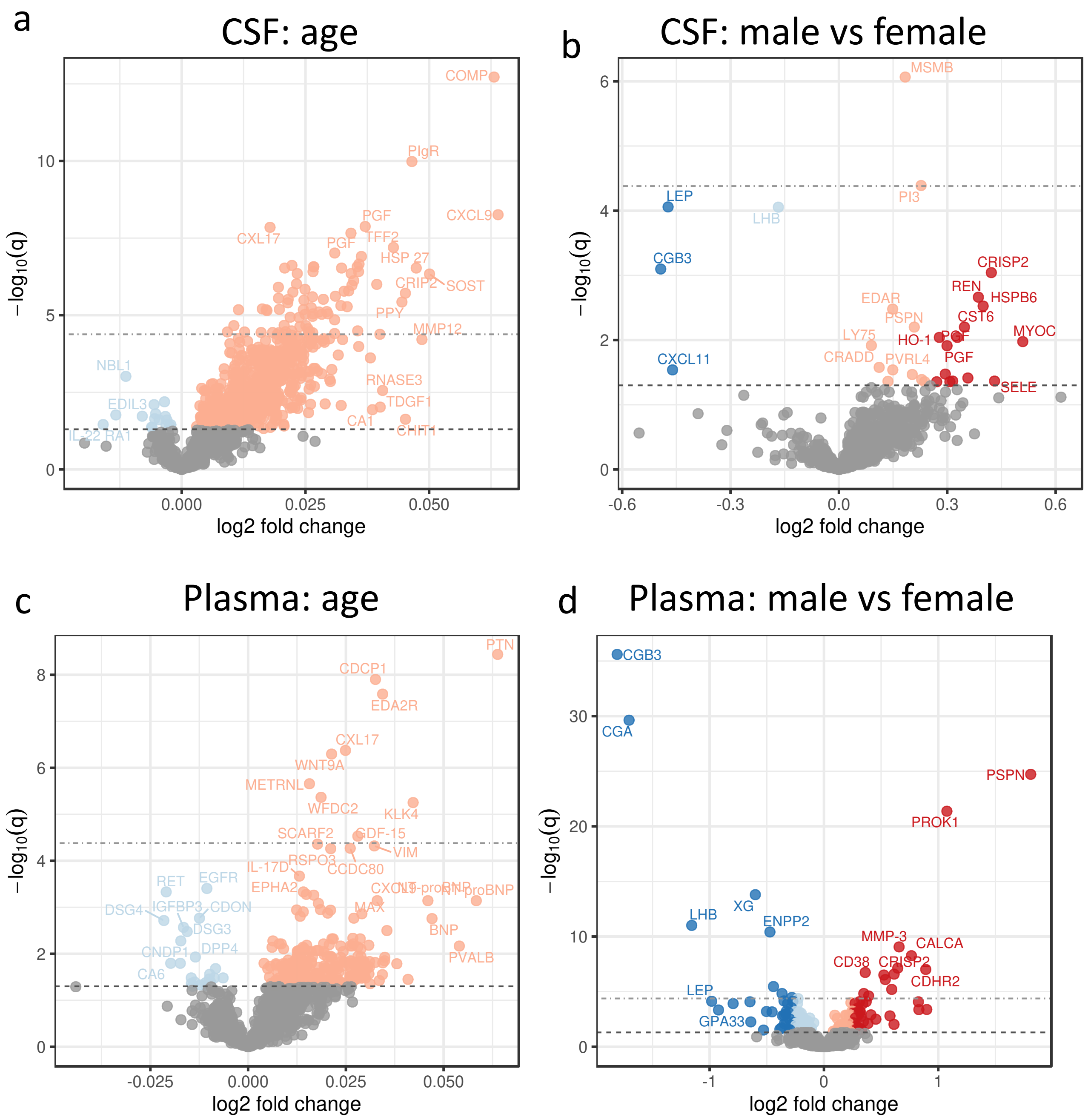

Supplementary Figure 2. Differential expression of proteomics in the Stanford cohorts for Age and Gender. Lower horizontal dotted line indicates FDR significance threshold, upper horizontal line indicates Bonferroni significance threshold. Dotted vertical lines indicate an arbitrary 0.25 log2 fold change cutoff for differential expression. Significant hits are colored and shaded by cutoff. **a,b)** CSF expression. **c,d)** Plasma expression.

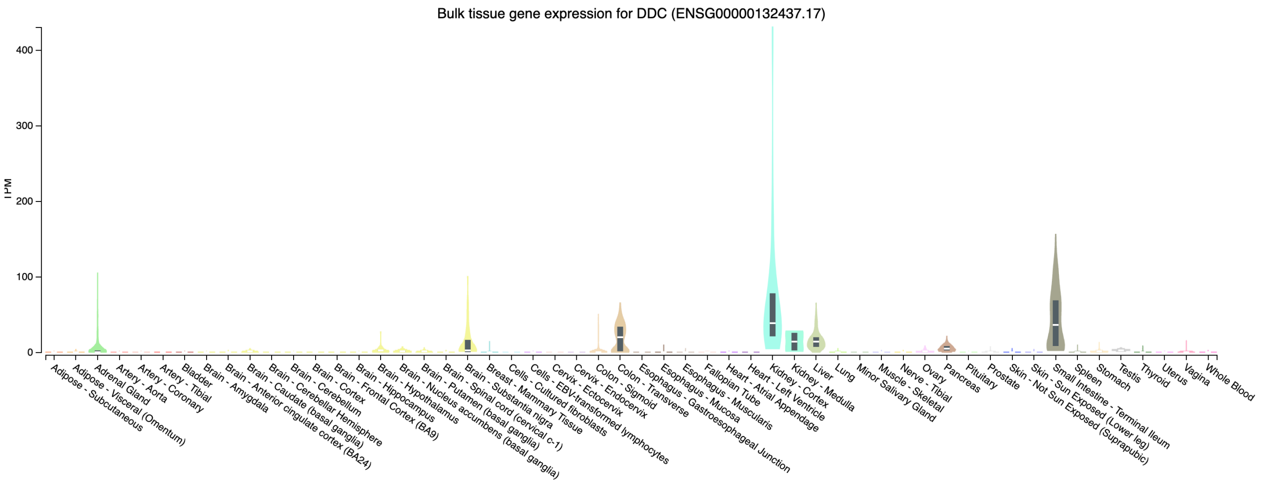

Supplementary Figure 3. Tissue RNA expression level of the DDC gene in the human Genotype Tissue Expression (GTEx) resource. DDC is highly expressed in the substantia nigra of the brain compared to other brain regions.

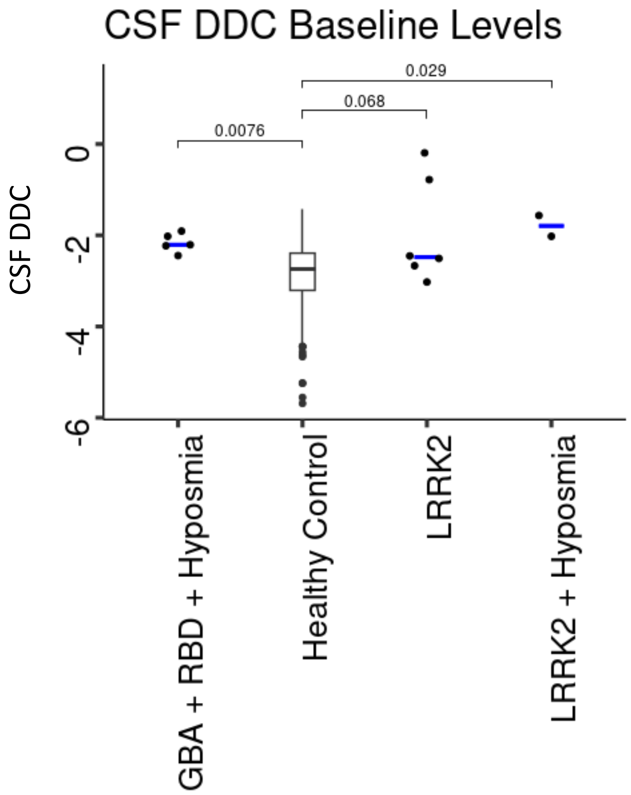

Supplementary Figure 4. DDC is elevated in CSF of non-manifesting GBA and LRRK2 carriers in the PPMI cohort. Blue lines represent median values. Box is shown for Healthy Control because of much larger sample size.

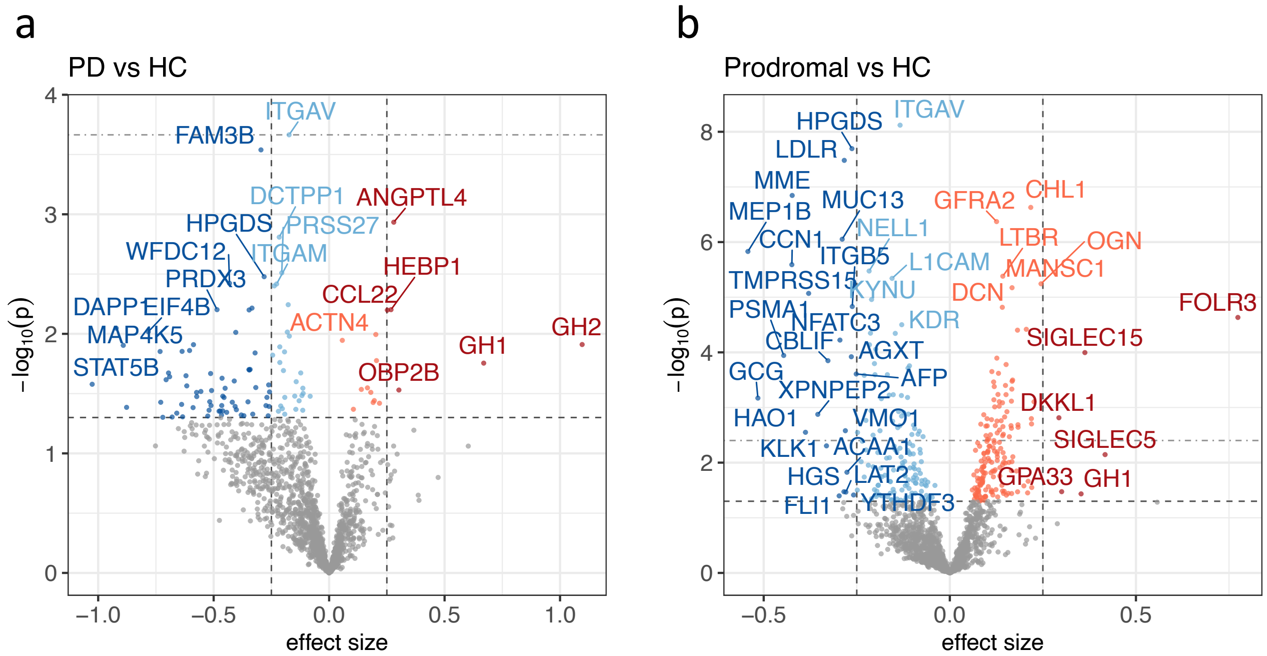

Supplementary Figure 5. Proteome-wide differential expression analysis of plasma samples from the PPMI 1 cohort using Olink proteomics. Top horizontal dotted line indicates FDR significance threshold, bottom horizontal dotted line indicates raw p-value threshold. Dotted vertical lines indicate an arbitrary 0.25 effect size cutoff for moderate-effect proteins. Significant hits are shaded by effect size cutoff. **a)** Treatment-naïve PD vs HC analysis. **b)** Prodromal PD vs HC analysis.

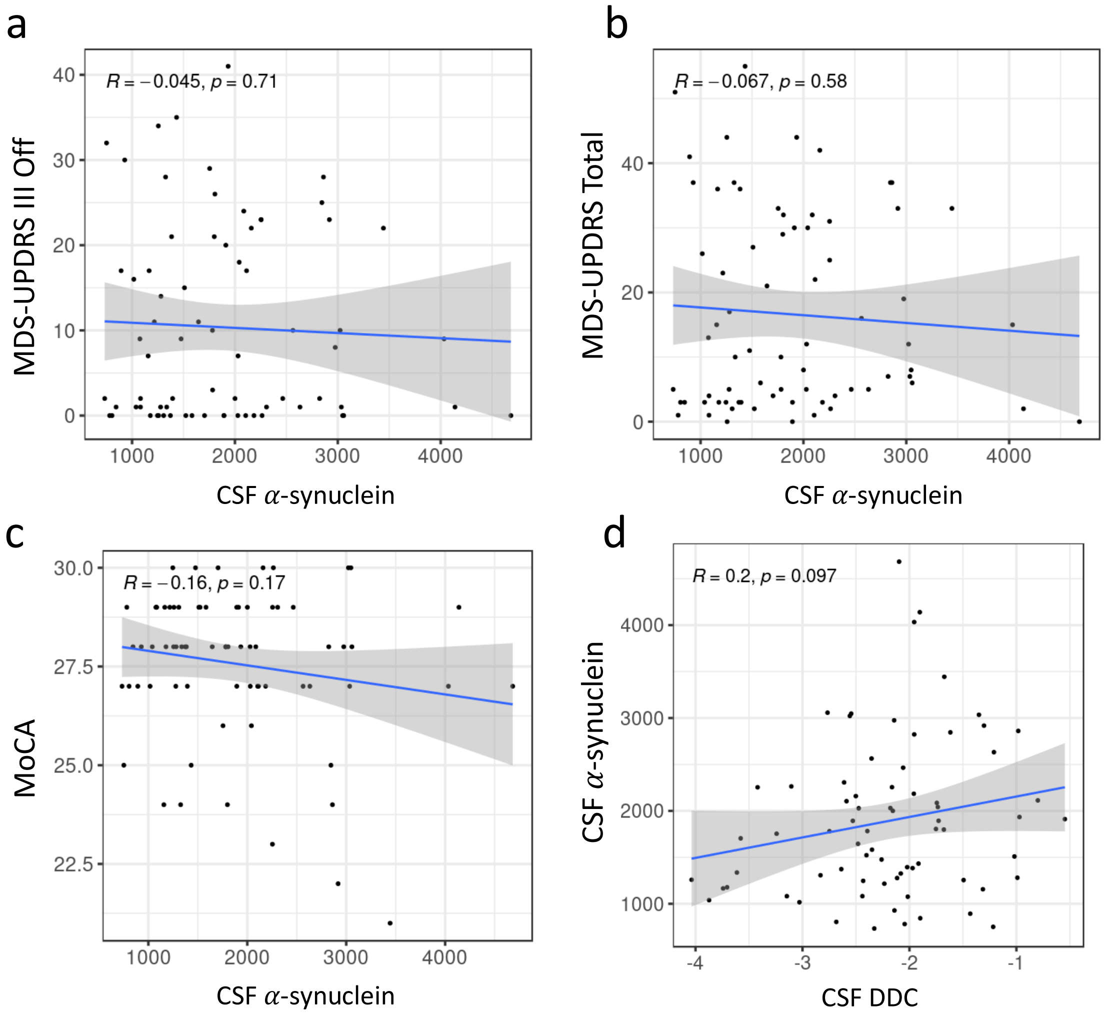

Supplementary figure 6. CSF alpha synuclein level is not associated with motor or cognitive symptoms at baseline in the treatment-naïve PPMI cohort. **a)** MDS-UPDRS III, clinician-assessed motor symptoms. **b)** MDS-UPDRS total symptom score. **c)** MoCA score. **d)** Correlation between CSF ADC levels at baseline in treatment naïve PPMI cohort and CSF alpha synuclein levels.

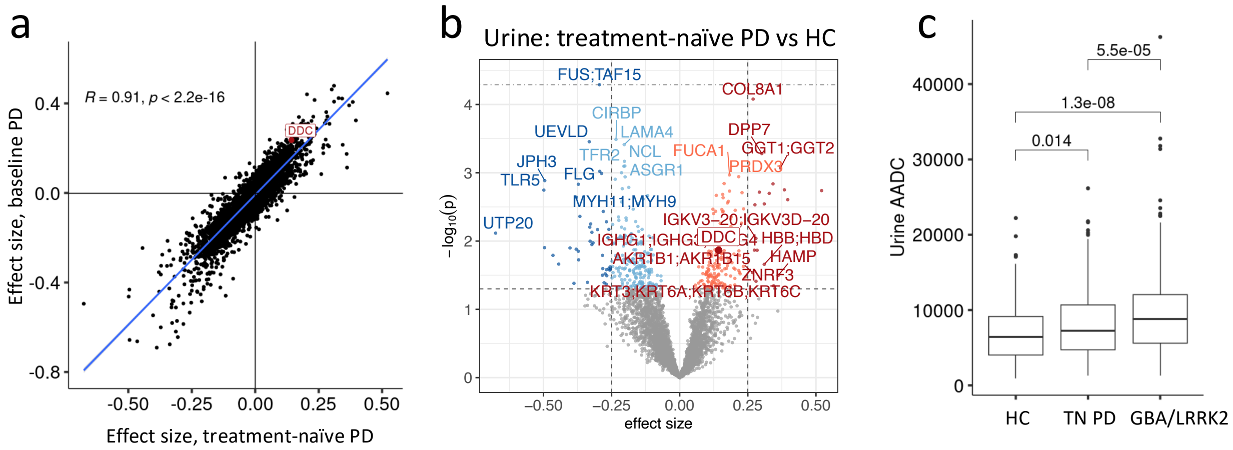

Supplementary Figure 7. Strong overlap between baseline PD and treatment-naïve PD effects in PPMI 2 urine proteomics. **a)** Effect sizes in differential expression are strongly correlated between baseline and treatment-naïve samples, including for DDC (highlighted). **b)** Volcano plot of treatment-naïve baseline PD vs HC differential expression analysis. Top hits are preserved. **c)** DDC levels between HC, treatment-naïve PD (TN PD) and non-manifesting GBA or LRRK2 carriers (GBA/LRRK2).
